# Effects of Electroconvulsive Therapy on Depression and Its Potential Mechanism

**DOI:** 10.3389/fpsyg.2020.00080

**Published:** 2020-02-20

**Authors:** Ming Li, Xiaoxiao Yao, Lihua Sun, Lihong Zhao, Wenbo Xu, Haisheng Zhao, Fangyi Zhao, Xiaohan Zou, Ziqian Cheng, Bingjin Li, Wei Yang, Ranji Cui

**Affiliations:** Jilin Provincial Key Laboratory on Molecular and Chemical Genetics, The Second Hospital of Jilin University, Changchun, China

**Keywords:** depression, ECT, neuroplasticity, Homer1a, cognition

## Abstract

Depression is one of the most common disorders causing mortality around the world. Although electroconvulsive therapy (ECT) is, along with antidepressants and psychotherapy, one of the three major treatments of depression, it is still considered as the last resort for depressed patients. This situation is partially due to limited studies and uncertainty regarding its mechanism. However, decades of increased research have focused on the effects of ECT on depression and its potential mechanism. Furthermore, these investigations may suggest that ECT should be a first-line therapy for depression due to its profound effects in relieving desperation in certain situations. Here, we outline recent clinical and preclinical studies and summarize the advantages and disadvantages of ECT. Thus, this review may provide some hints for clinical application.

## Introduction

Depression, the most prevalent mood disorder worldwide, has been indicated as an increasing social burden and as causing a significant proportion of mortality. Depression, occurring beyond or around and even among us, is the most common mental illness. Several hypotheses have been proposed for the cause of depression (Malhi and Mann, 2018). However, no single hypothesis can explain the full disorganization of depression or why therapeutic responses demonstrate individual differences (Malhi and Mann, 2018). Currently, there are two main treatments to fight depression, antidepressants and psychotherapy, while a third approach, electroconvulsive therapy (ECT), is regarded as a second- or third-line therapy that is usually resorted to in cases where medication and psychotherapy have failed (Kellner et al., 2016b; Karayagmurlu et al., 2019). However, most patients who were resistant to antidepressant or psychotherapy showed improvement after ECT was introduced. In other words, ECT may have a greater effect than the two routinely used methods in fighting depression (Husain et al., 2004; Spaans et al., 2015; Liang et al., 2017, 2018). Furthermore, the rate of failure in medication or psychotherapy is high and consistent (Schoeyen et al., 2015; Malhi and Mann, 2018). Therefore, further knowledge on how to treat patients resistant to these treatments is urgently needed.

Electroconvulsive therapy is a procedure that applies electrical stimulation to produce a generalized seizure. Though first introduced more than half a century ago, ECT is reserved as the last resort in treating severe mental disorders, such as depression or bipolar disorder (Husain et al., 2004; Schoeyen et al., 2015; Malhi and Mann, 2018). The reason for this difference between ECT and other remedies is partly due to its unfavorable procedure and side effects. Indeed, compared with antidepressant drugs, induced seizures is required to deliver an electrical stimulus. Moreover, ECT recipients endure more headaches and muscle aches (Husain et al., 2004). However, when anesthesia is applied and improved apparatus, such as the unilateral device, is applied, ECT results are encouraging (Liang et al., 2018; Osler et al., 2018). Recently, extensive studies have suggested that ECT is highly effective in ameliorating depression symptoms with fewer unwanted effects (Liang et al., 2017, 2018; Karayagmurlu et al., 2019). Similar findings were also reported in animal studies using electroconvulsive seizures (ECS, an animal model for ECT) (Jonckheere et al., 2018; Alemu et al., 2019). Therefore, this review was aimed to interpret recent clinical and preclinical ECT studies and its potential mechanism on depression to provide clinicians and patients with greater appreciation of this approach to defeat depression.

## Searching Strategy and Selection Criteria

We searched PubMed for studies published between June 1, 2010, and June 1, 2018, using the terms “depression,” “depressive disorder,” and “depressive disorder, major,” with specifiers “therapy” as well as “ECT,” “electroconvulsive shock,” and “electroconvulsive seizure.” In addition, we retrospectively pooled studies conducted by the CORE/PRIDE Work Group. We only included publications written in English and focused on publications from the past 5 years. We retrieved full-length articles for all the publications selected.

## Clinical Studies of ECT

Upon its introduction, more than half a century ago, ECT has been applied to benefit patients in clinical application, especially for mental disorders (Husain et al., 2004; Liang et al., 2018; Osler et al., 2018). Generally, only if medication and psychotherapy are unsuccessful is ECT considered. ECT was generally underused. Compared with ECT without anesthesia, modified ECT was found to be more durable and even favored in some circumstances deemed treatment-resistant after several rounds of medication and psychotherapy. Indeed, when compared to medication, ECT may not display performance inferior to medication, and might even have superior effects (Husain et al., 2004; Nuninga et al., 2018; Omori et al., 2019). Therefore, based on recent clinical studies, we illustrate what has been done with ECT compared with antidepressants. Differences between symptom assessment, medical imaging, and biological studies before and after ECT was applied will be discussed in this section. Furthermore, coordinated symptom assessment and medical imaging studies and predictions of remission and relapse are discussed and will be interpreted in this section.

### Clinical Observation by Questionnaire Assessments

Electroconvulsive therapy is a favorable method to reduce depression symptoms regardless of whether antidepressants are involved (Husain et al., 2004; Kellner et al., 2016b). We first introduce some studies involving multi-site collaborations with relatively moderate or large samples. The Consortium for Research in ECT (CORE), supported by four collaborating clinical centers, showed a significant effect of bilateral ECT on unipolar depressed patients (Husain et al., 2004; Kellner et al., 2006). With 253 patients total enrolled without antidepressants, an acute series of ECT demonstrated a relatively high speed and rate response and remission (Husain et al., 2004). In addition, to prevent relapse, the CORE group conducted continuation ECT, indicating that continuation ECT can prevent remitted individuals from relapse and is non-inferior to combined pharmaceutical treatment (Kellner et al., 2006), and this effect seems to work regardless of racial differences (Williams et al., 2008). Of note, most CORE studies apply bilateral electrodes. Some studies sought to evaluate a unilateral electrode apparatus; the CORE group investigated the patients’ response to bifrontal, bitemporal, and right unilateral electrode placement in ECT in 230 depressed patients who were randomly assigned to one of the three electrode placements. While all three groups showed significant clinical improvement, the bilateral electrode may induce a more rapid response, whereas bitemporal may perform the best with respect to symptom amelioration (Kellner et al., 2010). Though ECT is comparatively safe, studies conducted in a restricted population had assorted memory impairment that could be ascribed to ECT, especially with long-term use. Thus, a comparison of memorial practice with 24-week follow-up between continuation ECT and continuation pharmacologic intervention showed no statistical difference in memory performance (Smith et al., 2010).

Additionally, owing to the work of the CORE group, which showed the equal performance of continuation ECT and combination pharmacotherapy in preventing relapse following response to acute ECT, the Prolonging Remission in Depressed Elderly (PRIDE) group was established. The PRIDE/CORE group was established to investigate whether combined pharmacotherapy and ECT, personalized according to patient reaction, is more effective in preventing relapse in depressed older individuals than pharmacotherapy alone. For the phase I study, right unilateral ultrabrief pulse ECT combined with venlafaxine was introduced for the treatment of geriatric depression, showing that right unilateral ultrabrief pulse ECT combined with venlafaxine worked quickly and effectively against geriatric depression, with excellent safety and tolerability (Kellner et al., 2016b). Phase II participants were recruited from the remitted individuals of the PRIDE phase I study, and phase II was conducted using a novel Symptom-Titrated Algorithm-Based Longitudinal ECT (STABLE) regimen. As a result, the continuation ECT plus medication was preferable in clinical performance and did not show statistically different cognitive impairment from that of continuation medication alone (Kellner et al., 2016a), and STABLE resulted in overall net health benefits (McCall et al., 2018). As for health-related quality of life in elderly depressed patients who underwent ultrabrief-pulse ECT, an open-label study suggested that attaining remission was critical to acquiring better health quality (McCall et al., 2017).

In short, the CORE/PRIDE studies found that ECT was not inferior to and possibly had more preferable outcomes than antidepressants in certain circumstances. Although a bilateral electrode apparatus may perform better than a unilateral one, application of a special algorithm made the unilateral apparatus easier to implement in clinical practice. Continuation ECT meaningfully maintained remission and, combined with medication, may maximize the benefits of ECT without increases in memory loss (Omori et al., 2019).

In addition to CORE/PRIDE, some studies have also investigated the profile of ECT based on a clinical questionnaire assessment as to whether ECT is more likely to be inferred through subjective differences or bioinformatic measures through medical imaging or laboratory methods.

As CORE demonstrated that elder depressed individuals may respond better to bilateral ECT (Husain et al., 2004), Spaans et al. (2015) suggest that geriatric depression also responds rapidly to unilateral ECT. A double-blind, randomized controlled trial of ultrabrief- and brief-pulse unilateral EC found no differences between the two groups, and that if high dosage was applied in an ultrabrief pulse, less cognitive impairment was seen (Loo et al., 2014), and this pattern may apply to bilateral ECT as well (Martin et al., 2019). Similarly, high-dosage brief-pulse unilateral ECT may not be inferior to bitemporal ECT based on the 24-item Hamilton Depression Rating Scale (HAM-D) score, while the unilateral ECT group showed a more favorable cognitive portrait (Semkovska et al., 2016). According to a 17-year retrospective study of 1571 ECT recipients, psychiatric individuals who received ECT had lower mortality than those who did not, though nested social and physical parameters affected the results (Liang et al., 2017), and another multistate observation study showed the effect of ECT in reducing short-term readmission risk among those with server affective disorders (Slade et al., 2017). With respect to effects on memory, Brus et al. (2017) used national register-based information to inquire about the rate of subjective memory worsening (SMW) reported with ECT, suggesting that patients who were female, young, less cognitively impaired before ECT, treatment-resistant, and experiencing wider pulse width were more likely to be subject to SMW, and their SMW reported rate was not low. However, this study had such limitations as significantly heterogeneous interference, subjective report collection, and the details of the ECT procedure. Furthermore, many patients referred to ECT may complain of short-term memory deterioration (Nuninga et al., 2018) or no significant alteration compared to treatment with antidepressants (Bjoerke-Bertheussen et al., 2018; Osler et al., 2018), and for those who were afflicted with cognitive decline before ECT, evidence of cognitive improvement was presented (Socci et al., 2018). In relation to suicide, large-scale nationwide studies performed retrospectively indicate that in patients with unipolar disorder and bipolar depression, ECT had superior anti-suicidal effects (Liang et al., 2018). However, for adolescent and young adult females with a history of non-suicidal self-injury, lower odds of response and remission and great mean times of treatment were observed (Rootes-Murdy et al., 2019).

Apart from non-CORE/PRIDE, the studies above indicate that older age, male, and with baseline cognitive decline were predictors of a preferable response and less likelihood of a relapse (Socci et al., 2018; van Diermen et al., 2018). Contrariwise, depressed individuals who were young, female, and with a history of non-suicidal self-injury were more likely to be resistant to ECT (Socci et al., 2018; Rootes-Murdy et al., 2019). In addition, ultrabrief may not be superior to brief-pulse ECT in symptom improvement, but memory disturbance was less reported (Bjoerke-Bertheussen et al., 2018; Martin et al., 2019). Overall, ECT may benefit depressed patients regardless of heterogeneous backgrounds.

### Clinical Observation by Medical Imaging Programs

Medical imaging programs are a powerful tool in clinical practice. Among these medical applications, magnetic resonance imaging (MRI) is more suitable for the measurement of ECT-related alteration. Regarding MRI, both structural and functional parameters were noted as useful for describing the profile of ECT. Therefore, we conducted MRI comparison at baseline and after ECT to illuminate the possible benefits of ECT. All the MRI studies were conducted in the last 5 years.

First, individuals with major depressive disorder (MDD) subject to brief-pulse bilateral ECT showed significant volume increases in the bilateral medial temporal cortices, inferior temporal cortices, and right anterior cingulate after ECT, and the increased ratio was correlated with the clinical improvement measured by the HAM-D (Ota et al., 2015). With respect to 12 treatment-resistant depressed patients who received brief-pulse bifrontotemporal ECT, bilateral medial temporal lobe and perigenual anterior cingulate cortex volume increases were archived after ECT, and left medial temporal lobe volume increase was associated with significant clinical improvement (Cano et al., 2017). The hippocampus, which plays an essential role in memory formation and emotional plasticity, was the primary focus of MRI changes. Small hippocampal volume at baseline predicted more profound symptom improvement, and the hippocampal and the amygdala volume increases with ECT were correlated with symptom improvement (Joshi et al., 2016). After a series of ECT with a predominately right placement of a unilateral electrode, the volume increases in the right hippocampal cornu ammonis (CA2/3), dentate gyrus (DG), and subiculum regions were correlated with depression reduction and the method of electrode placement (Abbott et al., 2014). In addition, the volume of the CA subfields, granule cell layer, molecular layer, and subiculum of the hippocampus increased in severe MDD patients, and is possibly attributable to neurogenesis (Cao et al., 2018). However, with 7.0 T MRI applied to detect hippocampal volume changes in MDD patients who underwent ECT, a large and significant increase was observed after ECT in only the DG of the hippocampal volume. Furthermore, the increase in DG volume was related to a decrease in depression scores (Nuninga et al., 2019). However, in a study of longitudinal MRI and clinical data from the Global ECT-MRI Research Collaboration (GEMRIC), the subcortical gray matter increase was found to be negatively associated with total ventricle volume, while total white matter volume remained unchanged after ECT. In particular, the gray matter volumetric enlargements may not be predictive of a favorable outcome, though this was partially attributed to the heterogeneity among patients and the procedure and apparatus used (Ousdal et al., 2019). Moreover, a retrospective study revealed that though gray matter was enlarged, the changes may not correlate with psychopathology, age, gender, or number of ECT sessions (Sartorius et al., 2019), as did a longitudinal study of severe late-life unipolar depression (Bouckaert et al., 2016).

Although there remain controversies on the role of gray matter enlargement in clinical improvement and outcomes, the increased volume of gray matters in specific regions was relatively significant. As for a structural analysis, a functional algorithm may favor the use of MRI when examining the application of ECT in depression.

With regard to functional analysis, the hippocampal region remained hot. Abbott et al. (2014) found that hippocampal connectivity was enhanced after a series of ECT, and this change seems to correlate with symptom improvement and depends on how the electrode was fixed and located. Resting-state networks, believed to be neuronal activities of the brain in resting phase, showed that in patients with severe and treatment-resistant unipolar depression, the dorsomedial prefrontal cortex (including the dorsolateral prefrontal cortex, orbitofrontal cortex, and posterior cingulate cortex) and anterior cingulate cortex (including the dorsolateral prefrontal cortex, sensorimotor cortex, parahippocampal gyrus, and midbrain) had a great potential to predict the possibility of recovery from depression after ECT use (van Waarde et al., 2015). In addition, an enhanced feedforward cortical–subcortical connectivity from the fusiform face area to the amygdala was observed in MDD patients who underwent series ECT (Wang et al., 2017). Moreover, Wang et al. (2018) demonstrated that ECT may contribute to enhanced interactions in the intra- and inter-networks in MDD patients that result in symptom alleviation. Employed with perfusion MRI, ECT contributed to hippocampal cerebral blood flow increases and declines in specific regions relevant to seizure physiology. This balance was based on built-in functional neuroplasticity (Leaver et al., 2019). The fractional amplitude of low-frequency fluctuations (fALFF) can serve as a measure of the relative contribution of low-frequency fluctuations within a specific frequency band to the whole detectable frequency range. Qiu et al. (2019) revealed that the fALFF of post-ECT patients in the cerebellum anterior lobe, fusiform gyrus, and parahippocampal gyrus tended to be normalized compared with the fALFF of pre-ECT patients.

In short, according to the functional MRI studies mentioned above, ECT studies have brought renewed interest to the neuroplasticity of the brain, not only the hippocampus, but neural networks also showed more profound activity (Cao et al., 2018; Wang et al., 2018; Nuninga et al., 2019; Qiu et al., 2019).

Clinical studies have been conducted to provide evidence and suggestions for clinical practice. With respect to what is suggested by the results of the work above, we discuss the safety of ECT and the predictions of response and remission in ECT used to provide instructions for depressed individuals.

## Safety and Effectiveness of ECT

Electroconvulsive therapy was shown to be a relatively safe method to treat depression and remedy treatment-resistant patients. Continuation ECT alone or continuation ECT combined with medication was favorable to remaining in a remission state after response to ECT. The CORE group revealed that ECT alone had rapid response and a high likelihood of remission with bilateral ECT in severe unipolar MDD patients (Husain et al., 2004) regardless of racial differences (Williams et al., 2008). In fact, the results of ECT exceeded those of medication of treatment-resistant bipolar depression as well (Schoeyen et al., 2015). Moreover, continuation ECT should be considered to prevent relapse (Kellner et al., 2006) without the cost of memory loss found with other treatments (Smith et al., 2010; Kellner et al., 2016a), and it was shown to have an overall net health benefit in older depressed individuals (McCall et al., 2018). In addition, besides depression, continuation ECT supported schizophrenia and schizoaffective disorder as well (Omori et al., 2019). As for electrode placement, bitemporal ECT showed rapid response, and unilateral placement may be inferior to bilateral in effects on depression (Kellner et al., 2010). However, high-dose unilateral ECT may not be inferior to bitemporal ECT and may achieve better cognitive performance (Semkovska et al., 2016). Additionally, regarding unilateral ECT, when proper dosage was applied, those receiving ultrabrief pulse suffered less cognitive decline than brief pulse without a difference in the effectiveness of depression alleviation (Loo et al., 2014; Martin et al., 2019). Moreover, the PRIDE Study showed that right unilateral ultrabrief-pulse ECT, combined with venlafaxine, performed with excellent safety and tolerability in treating geriatric depression (Kellner et al., 2016b) and improved health-related quality of life (McCall et al., 2017). In addition, ECT may reduce mortality among patients with psychiatric conditions (Liang et al., 2017) and short-term psychiatric inpatient readmissions whose symptoms were severe (Slade et al., 2017). In some urgent circumstances, suicide for example, ECT responded quickly and showed superior anti-suicidal effects in spite of unipolar or bipolar depression (Liang et al., 2018).

However, cognitive decline was noted in some depressed patients who received ECT (Brus et al., 2017), and recovery from this declination was suggested to take half a year (Nuninga et al., 2018). However, whether ECT contributed more memory loss than pharmaceutical treatment is still in dispute, but the majority support the view of no additional cognitive damage ascribable to ECT than antidepressants (Husain et al., 2004; Kellner et al., 2016a, b; Bjoerke-Bertheussen et al., 2018). Moreover, less cognitive withdrawal was seen in unilateral to bilateral (Semkovska et al., 2016) and ultrabrief pulse to brief pulse (Loo et al., 2014; Brus et al., 2017; Martin et al., 2019) when the dosage was properly applied. Nonetheless, some studies found no dementia in individuals who underwent ECT (Osler et al., 2018), and in geriatric depressed patients, ECT even improved cognitive function (Socci et al., 2018).

With respect to other common side effects that occurred during ECT treatment, headache and nausea/vomiting are believed to be the most common complaints (Kellner et al., 2006; Karayagmurlu et al., 2019). Thankfully, in the view of severity and prevalence, the ECT recipients reported less headache and nausea/vomiting in recent studies (Husain et al., 2004; McCall et al., 2018; Socci et al., 2018). Furthermore, headache and nausea/vomiting usually recovery in a few hours without medical intervention. Lastly, anti-symptom therapy is safe if necessary.

Briefly, ECT was found to be relatively safe and effective in treating depression and may be superior to medication in symptom improvement, especially for those who failed to recover after rounds of medication (Husain et al., 2004; Schoeyen et al., 2015; Spaans et al., 2015; Slade et al., 2017). Continuation ECT should be employed to prevent relapse if possible (Kellner et al., 2006, 2016a). Unilateral ultrabrief-pulse ECT may result in less cognitive declination when advanced procedures are applied (Semkovska et al., 2016). Controversies over cognitive decline following ECT may partially be due to heterogeneous factors, such as aging and quantitative measurements. More studies should be conducted to investigate the relationship between ECT and memory plasticity.

### Prediction of Response and Relapse to ECT, Bioinformatics

Comprehensive bioinformatics is assumed to be able to predict who is likely to benefit from ECT. It has been suggested that unipolar depressed patients with psychotic symptoms were more likely to respond to ECT and the elderly were more likely to reach remission (Husain et al., 2004; Socci et al., 2018; van Diermen et al., 2018). Additionally, the Maudsley Staging Method confirmed that shorter episode duration and more severe depressive symptoms predicted a favorable outcome (van Diermen et al., 2018). As a result of the overall prevalence of female depressed individuals, the number of female depressed patients who participated in ECT was greater than male (Liang et al., 2017; Slade et al., 2017; McCall et al., 2018). Interestingly, female ECT recipients may respond better in the postpartum period (Rundgren et al., 2018). In addition, estradiol withdrawal could be partially to blame, since estradiol has a protective role against depression in females (Schmidt et al., 2015) but boosts depression in young men (Stanikova et al., 2018). Furthermore, early improvement after two ECT sessions anticipated a final remission (Birkenhager et al., 2019). Based on structural and functional analysis, Abbott et al. (2014) suggested that increased hippocampal functional connectivity and volumes in MDD can predict the response to ECT, and functional-MRI-based resting-state networks predicted responses with great sensitivity and specificity (van Waarde et al., 2015). Moreover, the hippocampal subfield volumes at baseline anticipated clinical improvement when a machine learning algorithm was employed (Cao et al., 2018), and 7.0 T MRI indicated that the baseline DG may be a more specific predictor (Nuninga et al., 2019). In addition, a relatively small degree of structural impairment in the subgenual cingulate cortex seems to have responded strongly to ECT in a non-randomized prospective study (Redlich et al., 2016). In fact, with respect to treatment-resistant depressed individuals, ECT showed a rate of response and remission approaching that of antidepressants (Schoeyen et al., 2015). Njau et al. (2017) revealed that lower N-acetyl-L-aspartic acid (NAA) levels in the dorsal anterior cingulate cortex (dACC) at baseline predicted a better outcome for ECT recipients. Genetically, ECT recipients with high genetic risk load tended to be less responsive to ECT (Foo et al., 2019). On the other hand, homozygous catechol-*O*-methyltransferase (COMT) G/G genotype was more sensitive than mutant heterozygous genotype (A/G), as was Homer rs7713917 A > G (Benedetti et al., 2018). Lower promoter methylation rates of BDNF exon I were strictly associated with remission (Kleimann et al., 2015), and the CC genotype of BDNF polymorphism C270T contributed a useful response (Huuhka et al., 2007). In support of inflammation, higher levels of IL-6 at baseline anticipated profound depression relief, especially in females (Kruse et al., 2018), and the degree of change in serum matrix metalloproteinase-9 (MM-9) was associated with relapse following ECT (Shibasaki et al., 2018).

In short, in the elderly (Husain et al., 2004), psychotic symptom comorbidities (Husain et al., 2004), postpartum depression (Rundgren et al., 2018), shorter episode duration and more severe depressive symptoms (van Diermen et al., 2018), early depression alleviation (Birkenhager et al., 2019), lower NAA levels in the dACC at baseline (Njau et al., 2017), low genetic risk load (Foo et al., 2019), genetic or epigenetic modifications (Huuhka et al., 2007; Kleimann et al., 2015; Lin et al., 2015), and high baseline serum IL-6 levels (Kruse et al., 2018) and a serum MM-9 profile (Shibasaki et al., 2018) were more likely to benefit ECT recipients. Moreover, the subfield index of hippocampus and neural network plasticity (Abbott et al., 2014; Ota et al., 2015; van Waarde et al., 2015; Redlich et al., 2016; Cao et al., 2018; Nuninga et al., 2019) may provide precise evidence to predict the outcome. In addition, to the best of our knowledge, no study has reported an obvious discrepancy regarding the effectiveness of ECT between genders, as sex hormones may be linked with depressive formation, for estradiol-protected women with past perimenopausal depression (Schmidt et al., 2015) while exacerbating depressive symptoms in young males (Stanikova et al., 2018).

Here, we introduce the latest, crucial clinical studies, CORE/PRIDE for example, to demonstrate the profile of ECT utilization in clinical practice. We summarize the recent and major clinical studies in Table 1, which includes studies involving questionnaire assessment, medical imaging analysis, effectiveness and safety assessment, and bioinformatics for the prediction of response and relapse. According to the clinical observation studies by questionnaire assessment, ECT was a very useful tool to counteract depression and alleviate depressive symptoms. Continuation and advanced ECT was better able to maximize benefits and minimize side effects. Based on medical imaging programs, the structural and functional plasticity of the brain, especially the limbic system, was noted and probably contributed to symptom improvement. Assumedly, this neuroplasticity resulted from neurogenesis and is associated with ECT response and effectiveness. As for its safety and effectiveness, not only can ECT improve depressive symptoms, but its effects may be superior to those of classical psychotherapy and antidepressants in exclusively treatment-resistant depression. However, evidence of cognitive impairment is not conclusive; ECT was still preferable in improving life quality, memory decline was not exclusive to ECT and was worse with antidepressants (Bjoerke-Bertheussen et al., 2018), and cognitive status was restored in the short term (Nuninga et al., 2018). In addition, for the depressed patients who had cognitive impairment at baseline, ECT may improve cognitive performance (Kellner et al., 2006; Osler et al., 2018). However, there were several social and physical factors, medical insurance coverage and physical tolerance for example, that impede patients in receiving ECT. Therefore, ECT is a very useful tool to counter depression and should not be perceived as the last line for depressive treatment (Husain et al., 2004; Liang et al., 2017).

**TABLE 1:** Summary of major ECT clinical studies.

**Study design**	**Parameters**	**Major findings**	**References**
TRD + previous medication + a-ECT ± c-ECT	Right unilateral + unknown	Increased BDNF and not significantly correlated with clinical improvement	Vanicek et al., 2019
TRD + previous medication + a-ECT ± c-ECT	Bifrontotemporal + brief pulse	Use of mood stabilizers and maintenance ECT to prevent relapse	Omori et al., 2019
MDE + a-ECT	Bitemporal + brief/ultrabrief pulse	Equal antidepression, better cognitive performance for ultrabrief pulse	Martin et al., 2019
MDE + a-ECT	Bitemporal + brief pulse	Early symptoms improvement predicted quicker remission	Birkenhager et al., 2019
MDE + a-ECT + MRI	Bilateral/unilateral + brief pulse	Gray matter volume increases not correlated with clinical improvement	Sartorius et al., 2019
MDD + a-ECT + MRI	Bitemporal + brief pulse	Enhanced functional plasticity in specific brain regions (normalized fALFF)	Qiu et al., 2019
Depression + MRI	Bifrontotemporal + unknown	Increase in DG volume, correlated with clinical improvement	Nuninga et al., 2019
MDD + a-ECT + polygenic risk score	Right unilateral + brief pulse	High polygenic risk score anticipated poor outcome	Foo et al., 2019
MDD + antidepressants + a-ECT + MRI	Bifrontal + unknown	Enhanced intra- and internetwork plasticity within the response	Wang et al., 2018
Depression + previous medication + a-ECT	Bilateral/unilateral + brief pulse	Shorter episode duration, more severe depression, and older age predicted ECT effectiveness	van Diermen et al., 2018
Postpartum depression and psychosis + ECT	Bilateral/unilateral + unknown	Postpartum depression and psychosis responded greatly to ECT	Rundgren et al., 2018
MDD + ECT + MRI	Bilateral/unilateral + unknown	Hippocampal volume increases not related with symptom improvement, greater increased indicated poor outcome	Oltedal et al., 2018
Depression + a-ECT/c-ECT	Bifrontotemporal + unknown	Bilateral ECT showed cognitive decline that recovered in 6 months	Nuninga et al., 2018
MDD + c-ECT	Right unilateral ultrabrief pulse	Overall net health benefit	McCall et al., 2018; Rundgren et al., 2018
TRD + a-ECT	Bilateral/unilateral + brief pulse	Baseline serum IL-6 level predicted response	Kruse et al., 2018
Severe MDD + a-ECT + MRI	Bilateral + unknown	Machine learning algorithm to the hippocampal subfield volumes at baseline to predict response	Cao et al., 2018
MDD + previous medication + a-ECT + MRI	Bifrontal + unknown	Enhanced the feedforward cortical subcortical connectivity from FFA to amygdala	Wang et al., 2017
Unipolar/bipolar depression + a-ECT + MRI	Unilateral + ultrabrief pulse/bilateral + brief pulse	Lower baseline NAA in the dACC predicted favorable outcome	Njau et al., 2017
Older unipolar MDD + previous therapy + a-ECT	Unilateral + ultrabrief pulse	Health-related quality of life improved regardless of cognitive impairment in short term	McCall et al., 2017
TRD + previous medication + a-ECT + MRI	Bifrontotemporal + brief pulse	Left MTL volume increase associated with hippocampal NAA decrease, Gla + Gln increase, and clinical improvement	Cano et al., 2017
Depression + a-ECT	Bitemporal/high dose unilateral + brief pulse	Non-inferior high dose unilateral and less cognitive decline	Semkovska et al., 2016
Depression + antidepressants + a-ECT + MRI	Bilateral/unilateral brief pulse	Small degree of structural impairment baseline in the subgenual cingulate cortex predicted better outcome	Redlich et al., 2016
Remitted a-ECT patients + c-ECT + medication	Unilateral + brief pulse	c-ECT + venlafaxine + lithium surpassed venlafaxine + lithium only	Kellner et al., 2016a
MDE + previous therapy + a-ECT	Unilateral + ultrabrief pulse	a-ECT + venlafaxine to effectiveness and safety	Kellner et al., 2016b
Depression + a-ECT	Bifrontal/bitemporal/unilateral + unknown	Bitemporal electrode responded quicker	Kellner et al., 2010
Remitted unipolar MDD patients + c-ECT	Bilateral + unknown	c-ECT equal to antidepressants to prevent relapse	Kellner et al., 2006
MDD + a-ECT	Bilateral + unknown	Rapid response and remission of ECT	Husain et al., 2004

*MDD, major depressive disorder; MED, major depressive episode; TRD, treatment-resistant depression; ECT, electroconvulsive therapy; a-ECT, acute ECT; c-ECT, continuation ECT, NAA: N-acetyl-aspartate; MRI, magnetic resonance imaging; BDNF, brain-derived neurotrophic factor; fALFF, amplitude of low frequency fluctuations; FFA, fusiform face area; DG, dentate gyrus; dACC, dorsal anterior cingulate cortex; MTL, medial temporal lobe.*

### ECS in Preclinical Studies

ECS is an experimental animal model of ECT (Jonckheere et al., 2018). ECS successfully improved depression- or stress-associated performance by preventing depression- and stress-induced damage (Kyeremanteng et al., 2014; Luo et al., 2015; Schloesser et al., 2015; Jonckheere et al., 2018; Alemu et al., 2019). Notwithstanding the heterogeneity of clinical ECT research, major animal studies supported that ECS can induce neurogenesis, and the neuron or glial cell loss or malfunction was compensated by neurogenesis (Kaastrup Muller et al., 2015; Schloesser et al., 2015; Jonckheere et al., 2018). In brief, ECS-induced neurogenesis was the primary mechanism to encounter depression.

Behavioral analysis was recorded to demonstrate depressing activities in animal models and behavioral changes after undergoing ECS. Rats treated with chronic unpredictable mild stress (CUMS), which caused them to display depression-like behavior, exhibited decreased sucrose preference percentage (SPP) and impaired performance on the open field test (OFT), forced swim test, novelty suppressed feeding test (NSF), and Morris water maze, and ECS inversely increased SPP and OFT activities (Luo et al., 2011, 2014; Zhu et al., 2015a, b; Gao et al., 2016; Zhang et al., 2016). Similarly, in the Wistar–Kyoto (WKY) rat strain, a genetic model displaying depression- and anxiety-like behaviors and working memory deficit, ECS improved psychiatric and memory behaviors (Kyeremanteng et al., 2014).

Monoamine malfunction, hypothalamic–pituitary–adrenal (HPA) axis dysfunction, inflammation, disturbed neuroplasticity, and neurogenesis are believed to cause depression (Malhi and Mann, 2018). With respect to ECS, regulation of the HPA axis, neuroplasticity, and neurogenesis were mostly demonstrated in the animal model of depression-like behavior. Typical depression displayed a hyperactive HPA axis profile. Regarding depression, increased cortisol level is critical (Grinevich et al., 2012). Typical depression exhibited increased levels of adrenocorticotropic hormone (ACTH) and/or corticotropin-releasing hormone (CRH) without proper regulation by the hippocampus (Stetler and Miller, 2011). Additionally, downregulated glucocorticoid receptors were blamed to decrease the effectiveness of cortisol on target tissues (Zunszain et al., 2011; Chiba et al., 2012). The ineffectiveness of glucocorticoid inversely enhanced inflammation (Zunszain et al., 2011). Moreover, brain CRH was elevated following repeated ECS independent of depressive behavior. However, ECS did alleviate depressive symptoms (Kyeremanteng et al., 2014).

Regarding neurogenesis, adult hippocampal neurogenesis was essential to buffer stress responses and depressive behavior, while for neurogenesis-deficient mice, increased food avoidance in a novel environment after acute stress, increased behavioral despair in the forced swim test, and decreased SPP were shown compared with neurogenesis-normal mice (Snyder et al., 2011). In addition, Madsen et al. (2005) reported that ECS increased the number of new dividing cells in the frontal cortex, and the dividing cells were believed to be either oligodendrocytes or endothelial cells but not to express neurons. Furthermore, retrieved volume loss in the frontal cortex was compensated by cell proliferation (Madsen et al., 2005). The corticosterone-induced anxiety- and depressive-like behavior was rescued by the hippocampal neurogenesis of the adult-born neurons after ECS, while for the animals that were genetically deficient in neurogenesis of adult-born neurons, ECS cannot relieve behavioral deficiency. Thus, intact hippocampal neurogenesis was required for ECS to confront depression (Schloesser et al., 2015). Additionally, neuroplasticity triggered or enhanced by ECS administration significantly increased the number of mitochondria, synapses, and length of microvessels in depressed rats regardless of response (Chen et al., 2018). Employed with MAP6 KO mouse, a genetic model of depression, Jonckheere et al. (2018) demonstrated that synaptogenesis and neurogenesis compensated for behavioral deficits. ECS enhances the proliferation of adult hippocampal neuronal progenitors, and continuation ECS led to persistent behavioral and biological improvements. Moreover, volume increases in the hippocampus were specific after ECS applied to the animal model of depression-like behavior (Alemu et al., 2019). On the other hand, BDNF is a neurotrophin related to canonical nerve growth that plays a critical role in neurogenesis and neuronal plasticity. The expression of BDNF was significantly elevated in animal models of depression-like behavior after undergoing ECS (Zhang et al., 2016; Chen et al., 2018; Jonckheere et al., 2018). The effect of confronting depression needed to incorporate studies of mitochondria and synapses (Chen et al., 2018). However, in a genetic animal model of depression, though brain BDNF increased instantly after ECS was applied, it normalized after repeated BDNF. Contrariwise, repeated ECS consistently increased brain BDNF level in depression-free animals (Kyeremanteng et al., 2014). In conclusion, ECS has the ability to increase the levels of BDNF, though depression may mitigate against increases in BDNF.

With respect to cognitive performance, based on the CUMS animal models that reproduced depression, ECS deteriorated cognitive performance in animal models via (1) downregulating the ratio of hippocampal glutamate (Glu) and γ-aminobutyric acid (GABA) levels by promoting excessive expression of glutamic acid decarboxylase 65 (GAD65) (Luo et al., 2011); (2) dysregulating hippocampal synaptic plasticity, specifically downregulating long-term potentiation (LTP), postsynaptic density-95 (PSD-95), and phospho–response element binding protein (p-CREB) protein expression (Luo et al., 2014); (3) inducing inflammatory cytokine-mediated glutamate uptake dysfunction in the hippocampus (Zhu et al., 2015a); (4) promoting neuroinflammation and increasing the levels of Aβ1–40 and Aβ1–42 in the hippocampus (Zhu et al., 2015b); (5) dysregulating the NMDA receptor subunit 2B (NR2B)-(extracellular signal-regulate kinase) ERK-signaling pathway (Gao et al., 2016); and (6) up-regulating the ratio of the precursor of brain-derived neurotrophic factor (proBDNF)/mature BDNF (mBDNF) (Zhang et al., 2016). However, ECS caused different memory indexes between types of animal models (Luo et al., 2015). Moreover, contrary to other CUMS cognitive performance, ECS improved memory function in CUMS compared with the baseline cognitive index (Luo et al., 2015). However, ECS under anesthesia with propofol, dexmedetomidine, or ketamine was able to rescue ECS-exacerbated dysfunction to improve memory performance (Luo et al., 2011, 2014; Zhu et al., 2015a, b; Gao et al., 2016; Zhang et al., 2016). In spite of the effectiveness of anesthesia plus ECS in enhancing cognitive performance without undermining ECS effectiveness, modified ECT caused less cognitive concern in clinical studies, and even older depressed patients with cognitive decline retrieved memory after undergoing ECT (Osler et al., 2018; Socci et al., 2018). Moreover, modified ECT, ECT programmed with anesthesia, is commonly applied in clinical procedures and is widely used (Kellner et al., 2006, 2016a,2016b; Semkovska et al., 2016; Bjoerke-Bertheussen et al., 2018), but anesthetic was not deemed essential to prevent memory loss because modified ECT still conceived cognitive impairment (Kellner et al., 2010; Loo et al., 2014; Bjoerke-Bertheussen et al., 2018). However, there was no comparison of cognitive characters between pure ECT and ECT programmed with anesthesia. Thus, it is unknown whether anesthetic in fact aids against ECT-induced cognitive impairment.

Recent research has investigated the profile of ECS. As shown in Table 2, ECS alleviated depression-like symptoms in general, and the major animal models worked on were CUMS, and among models, memory changes may vary.

**TABLE 2:** Effect of ECS on depression in the preclinical studies.

**Animal**	**Model**	**Test**	**Results**	**References**
Male Wistar rats	Chronic restraint stress	FST	Normalized the volume of the hippocampal hilus to control levels; Normalized depression-like behavior in the FST.	Alemu et al., 2019
MAP6 KO model	Genetic	FST; NSF	Decrease the immobility time; increased the time spent climbing; Increased latency to eat.	Jonckheere et al., 2018
Male albino Swiss mice	Forced swim test	FST	Decreased the immobility time.	Socala et al., 2017
Adult male Sprague–Dawley rats	CUMS	SPT; MWM	Increased the percentage of sucrose preference, the total distance traveled, and the frequency of rearing.	Li et al., 2016
Male Sprague–Dawley rats	CUMS	SPT; MWM	Increased the values of SPP; Decreased space exploration time.	Zhang et al., 2016
Adult Sprague–Dawley rats	rECS	MWM	Increased the time reach the platform; Decreased the staying time and crossing time.	Zhu et al., 2015b
Adult male Sprague–Dawley rats	CUMS	SPT; MWM	Decreased the SPP of the rats with CUMS; Increased the SPP values of the rats.	Zhu et al., 2015a
Adult male Wistar rats and WKY rats	CUMS	SPT; MWM	Impaired WKY rats’ memories but improved CUMS rats’ memories; Elevated hippocampal BDNF and CREB proteins only in CUMS rats.	Luo et al., 2015
Adult male Wistar rats	CUMS	SPT; MWM	Exacerbated the memory damage; When administered in modified ECS, propofol improved memory.	Luo et al., 2014

*MFB, medial forebrain bundle; DG, dentate gyrus; UPLC, ultra-performance liquid chromatography; FST, forced swim test; NSF, novelty suppressed feeding test; SPT, sucrose preference test; SPP, sucrose preference percentage; MWM, Morris water maze; CUMS, chronic unpredictable mild stress; rECS, Repeated electroconvulsive shock. MDD, major depressive disorder; MED, major depressive episode; TRD, treatment-resistant depression; ECT, electroconvulsive therapy; a-ECT, acute ECT; c-ECT, continuation ECT; NAA, N-acetyl-aspartate; MRI, magnetic resonance imaging; BDNF, brain-derived neurotrophic factor; fALFF, amplitude of low frequency fluctuations; FFA, fusiform face area; DG, dentate gyrus; dACC, dorsal anterior cingulate cortex; MTL, medial temporal lobe.*

## The Potential Mechanism of ECS

With regard to the mechanism of ECS on neurogenesis, the increased volume of specific regions of the brain with the application of ECS has been demonstrated. This increase was associated with improved behavior and neuroplasticity (Madsen et al., 2005; Kyeremanteng et al., 2014; Luo et al., 2015). Homer-1, or homer protein homolog 1, consists of two major splice variants, short-form (Homer1a) and long-form (Homer1b/c) (Shiraishi-Yamaguchi and Furuichi, 2007). Homer1 is widely expressed in the central nervous system and constitutes a major part of the postsynaptic density. Homer1 links metabotropic glutamate receptors (mGluRs) and regulates their downstream pathway. Homer1a is an instant splice variant induced by neuronal activity to compete for mGluRs with long-term Homer1b/c. The balance between Homer1a and Homer1b/c determined neuronal plasticity: If Homer1a is dominant, the neurons show homeostatic plasticity, while neurons tended to be activated when Homer1b/c was dominant (Shiraishi-Yamaguchi and Furuichi, 2007; Hu et al., 2010). Moreover, Homer1 is predominantly located in the CA1 region of the hippocampus. Homer1a was transcriptionally induced only upon neuronal stimulation, as in seizures, for example (Shiraishi-Yamaguchi and Furuichi, 2007; Kaastrup Muller et al., 2015). Elevated Homer1a in the hippocampus enhances α-amino-3-hydroxy-5-methyl-4-isoxazolepropionic acid (AMPA) receptor clustering and its synaptic transmission to aggregate the AMPA receptor-dependent excitatory postsynaptic potential (EPSC) without altering presynaptic glutamate release (Hennou et al., 2003). Moreover, Homer1a regulates the mGluR-IP3 signaling pathway and induces hyperpolarization in pyramidal neurons to establish reduced excitability and maintain homeostatic neuroplasticity (Hu et al., 2010). In general, Homer1a dominantly provides negative signals as feedback to enhanced excitivity of neurons. In agreement with region-specific theory, adaptive changes to chronic stress and morphological discrepancies are observed compared with normal animals and are attenuated following ECS (Kaastrup Muller et al., 2015). ECS-induced Homer1a helped to rescue the reductions in the total length of apical dendrites and the number of apical terminal branches of CA3c neurons. The balance between Homer1a and spinophilin contributed to the molecular compensation induced by ECS (Kaastrup Muller et al., 2015). Homer1a caused a short disruption in mGluR1/5 and Homer1b/c complex during its relatively short effect. The plasticity of the ratio of Homer1a/1b/c exhibited different neuronal activities in the respective regions and responded to different stimulations. For example, in reaction to chronic stress, though Homer1b/c overexpressed, its coupling with mGluR1/5 was reduced in the hippocampus. Initially, stress induced pro-activated AMPA receptors and decreased the activity of mGluRs. It has been reported that enhanced expression of Homer1a in the medial prefrontal cortex (mPFC) demonstrated an antidepressant effect, while decreased Homer1a enhanced depressive-like behavior (Serchov et al., 2015). Contrariwise, overexpression of Homer1a in the hippocampus promoted vulnerability to stress (Kaastrup Muller et al., 2015). Additionally, Homer1 regulated the HPA axis independent of mGluR1/5. Homer1a modulated mGluR1/5-mediated excitatory postsynaptic currents and interacted with the NMDA receptor to produce a rapid-acting antidepressant effect (Wagner et al., 2015). Homer1a expression is the final way of mediating the antidepressant effects of different antidepressant treatments. Though Homer1a counteracts Homer1b/c, the role of the different splice variants provided different ways of internal environment changes. However, questions remain: What is the upstream signal applied to Homer1? What is the profile of the receptor distribution of mGluR1/5 and AMPA in subsequent neuroplasticity? What causes the discrepancy of memory behavior between animals and depressed patients? Though mGluRs activated neurogenesis, the levels of BDNF and the role of BDNF after ECS or ECT are still matters of intense debate.

**FIGURE 1:**
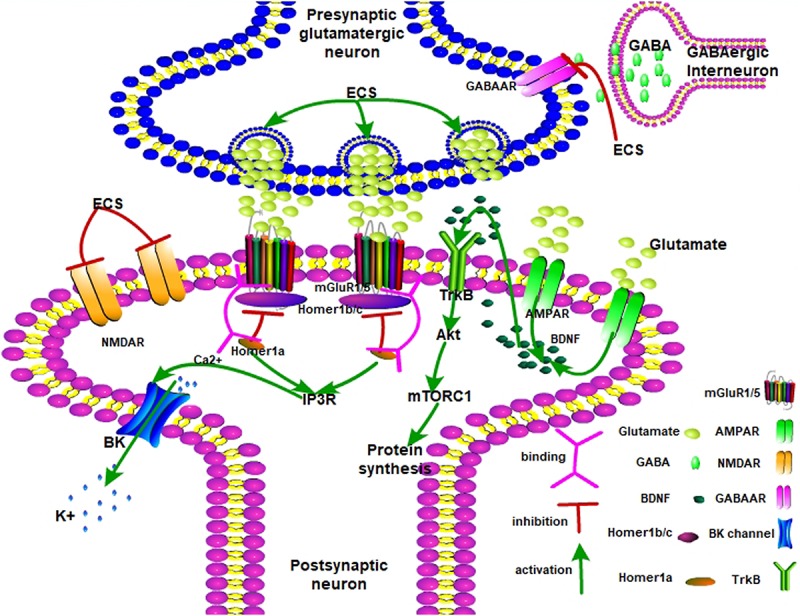
The possible antidepressant mechanisms of ECS on depression. AMPA receptor, α-amino-3-hydroxy-5-methyl-4-isoxazolepropionic acid receptor; mGlu1/5 receptor, metabotropic glutamate receptor 1/5; mTORC1, mammalian target of rapamycin complex 1; NMDAR, N-methyl-D-aspartate receptor; GABAAR, gamma-aminobutyric acid type A receptor; TrkB, tropomyosin receptor kinase B; Akt, protein kinase B; mTORC1, mammalian target of rapamycin complex 1; IP3R, inositol 1,4,5-trisphosphate receptor; BDNF, brain-derived neurotrophic factor.

Therefore, Figure 1 shows that ECS remodels neuroplasticity by mediating the balance between mGluR1/5 and AMPA receptors. ECS induced a fast-response antidepressant effect. Upon ECS application, the presynaptic glutamatergic neurons are activated while GABAergic neurons are inhibited. Glutamate is then released into the synaptic cleft and AMPAR is activated. Meanwhile, NMDAR is inhibited. Later, AMPAR releases BDNF to interact with TrkB. Next, activated Akt passes the signal to mTORC1 and promotes neurogenesis. On the other hand, Homer1 disrupts dysfunctional Homer1b/c and mGluR1/5 complexes and partially opens the BK channel by IP3R-released Ca^2+^. The BK channel leads the hyperpolarization of the postsynaptic neuron to present the antidepressant effect.

## Discussion

With regard to clinical utilization of ECT, many studies favor its effectiveness and relative safety. ECT had been introduced to treat diseases for decades, leading more researchers to investigate modified current ECT and explore its clinical indication. Without question, ECT alleviated depressive symptoms. Previously, ECT is mainly applied to treatment-resistant depressed patients, though it is also employed for other mental disorders. ECT successfully improved some patients’ symptoms independent of antidepressants (Husain et al., 2004; Loo et al., 2014), while when incorporated with certain antidepressants, sedatives for example, they mutually enhanced their antidepressive effects (Wang et al., 2018). Compared with ECT, continuation ECT is recommended for its ability to prevent relapse in those who were remitted following ECT (Kellner et al., 2006, 2016b). With respect to cognitive characters before and after ECT, no evidence indicates that ECT induces greater cognitive decline than antidepressants do (Kellner et al., 2006; Bjoerke-Bertheussen et al., 2018; McCall et al., 2018; Nuninga et al., 2018). However, memory impairment is common among depressed patients (Malhi and Mann, 2018). However, no studies have confirmed differences in cognitive profiles between patients, partially due to the heterogeneity of the clinical demonstrations. Moreover, although more advanced protocols were introduced, bioinformatics yielded predictions of the response of ECT that are scattered and lack comprehensive measurements (Husain et al., 2004; Redlich et al., 2016; van Diermen et al., 2018; Birkenhager et al., 2019; Foo et al., 2019; Nuninga et al., 2019; Omori et al., 2019). Additionally, even though ECT has been shown to be harmless and effective, the application rate is still low and it is considered the last resort in treating depression (Liang et al., 2017; Osler et al., 2018). Therefore, ECT should be more broadly used in treating depression, and more research focusing on how to predict responses in respective patients and how to enhance ECT currently in use is needed.

On the other hand, recent ECS studies have devoted greater effort to determining the mechanism behind the antidepressant effect. Similarly, ECS decreased depression-like behaviors and rescued molecular adaptive alteration responses to depression (Madsen et al., 2005; Kyeremanteng et al., 2014; Schloesser et al., 2015). However, there were discrepancies among model strains and behavioral reactions to exogenous stimulation (Kyeremanteng et al., 2014). Moreover, no single model can mimic depression in patients, due to the not fully disclosed mechanism of depression. In brief, the mechanism of depression remains under investigation, and while the effects of ECS on depression have been partially revealed, much remains unknown.

## Author Contributions

LZ, WX, HZ, ZC, ML, and FZ wrote the first draft. XY, LS, and XZ made major revisions to the logic of this article. BL, WY, and RC participated in the discussion of the manuscript. RC provided the critical revisions. All authors approved the final version of the manuscript for submission.

## Conflict of Interest

The authors declare that the research was conducted in the absence of any commercial or financial relationships that could be construed as a potential conflict of interest.
